# Supplementary material for: Chromothripsis during telomere crisis is independent of NHEJ, and consistent with a replicative origin
Source: Genome Res. 2019 May;29(5):737–49. doi: 10.1101/gr.240705.118 (PMC6499312; doi:10.1101/gr.240705.118)
Supplement: Supplemental Material [file supp_gr.240705.118_Supplemental_file_1.zip › contigs/annotated_contigs/DB89/contig.3.DB89_length_669_mean_cov_4.70852017937.docx]

**DB89_length_669_mean_cov_4.70852017937**

```
AAGCTGCCACTGGAGAGCTGTCAACCTAAAATAATCTCACATGTTCTCAATTGAAGAACCCTCCCAGGAAATGGATGATCAAGGAAATG
 >chr13:68663593-68663906 - E=1e-173
ATTACTCTTAAAATAAAAGTGTTTTTTTGAGATTCTAAATAGATGGTGTGTCCATAGTCCTTTCTCATGTCATTTTCTCAGACCAGATC

ATGTCTTTATCAATTAAGGCATAGCTAAGTATATTCATATTGACATTAAAATATGAATAACACATAGAATCAGTTTTGACATAGTGTTC

ATAAATCAAGATTGGGTATCTTAGGGAGACCACGTTTCTAAATGAT|GGTTCAGCTGGCTGTTCTGCTGTCTCCCTTTGGTTCCTCATG
                                                >chr19:36376350-36376581 - E=3e-127
TGGCTGCAATCAACCTGAGGCCCAACTGGCCTGGAAATCCACAACACCTTCACTCACATGCCTGATAGCTCAGCTGAGATAATGGAGCT

TCCAGGAGTGGCCCAAGAGTCTTTCCATGCAGTCCAGGAGGGCAGGTGGATCTCTTAACTTCAGGTGGTGGCTCAGTGGTTCCAAGAAA

GAGAGATCCA|A|ATTAGTGATGATGTGTCCTATCTGCAGGTACAGGAAGAGAGCTAATGGGGCAGTTTAATATTTGACATCTTTATTT
           >chr13:68663467-68663593 - E=5e-61
TTATGCATGCTTTCCCATAACTTATACAGCCATTTCAAGATTTACAAAA
```
